# Mega primer-mediated molecular cloning strategy for chimaeragenesis and long DNA fragment insertion

**DOI:** 10.1042/BSR20160608

**Published:** 2017-03-02

**Authors:** Hui Zhang, Chang-Jun Liu, Hui Jiang, Lu Zhou, Wen-Ying Li, Ling-Yun Zhu, Lei Wu, Er Meng, Dong-Yi Zhang

**Affiliations:** 1 Research Center of Biological Information, College of Science, National University of Defense Technology, Changsha, Hunan 410073, China; 2 Key Laboratory of Protein Chemistry and Developmental Biology of the Ministry of Education, College of Life Sciences, Hunan Normal University, Changsha, Hunan 410081, China; 3 Beijing Institute of Pharmaceutical Chemistry, Beijing 102205, China

**Keywords:** DNA insertion, Mega Primer, Seamless cloning

## Abstract

Molecular cloning methods based on primer and overlap-extension PCR are widely used due to their simplicity, reliability, low cost and high efficiency. In this article, an efficient mega primer-mediated (MP) cloning strategy for chimaeragenesis and long DNA fragment insertion is presented. MP cloning is a seamless, restriction/ligation-independent method that requires only three steps: (i) the first PCR for mega primer generation; (ii) the second PCR for exponential amplification mediated by the mega primers and (iii) DpnI digestion and transformation. Most importantly, for chimaeragenesis, genes can be assembled and constructed into the plasmid vector in a single PCR step. By employing this strategy, we successfully inserted four DNA fragments (approximately 500 bp each) into the same vector simultaneously. In conclusion, the strategy proved to be a simple and efficient tool for seamless cloning.

## Introduction

Seamless cloning and gene fusion are efficient tools for protein engineering, protein functional studies, protein production, promoter and exon studies and genome manipulation [1]. With the development of next-generation sequencing, high-throughput (HTP) recombinant DNA technologies are urgently required for protein engineering and heterologous protein expression to study the cellular and biochemical functions of proteins/peptides. With the development of high-fidelity DNA polymerases and PCR technology, many innovative seamless cloning methods based on primers and PCR have dramatically promoted the efficiency of DNA manipulation. For example, enzyme-free cloning [2] utilizes a compatible set of tailed and non-tailed primers (eight primers) to generate DNA fragments and linear vectors with cohesive ends, which can be directly transformed into *Escherichia coli*. The method is restriction/ligation-independent and easy to perform; however, it still requires the careful design of several primers to ensure the successful amplification of fragments and linearized vectors. A more straightforward, efficient and reliable method to clone one fragment into a destination vector is restriction-free (RF) cloning [3] or overlap-extension cloning (OEC) [4]. In a similar method to QuickChange^™^ site-directed mutagenesis (Stratagene, CA 92037, U.S.A.), RF cloning inserts a complete gene without the introduction of unwanted extra sequences, such as restriction sites or recombinant sequences. In addition, the primer design is simple and only a pair of hybrid primers is required (5′-end of the primer complementary to the target gene and 3′-end of the primer complementary to the insertion site of the cloning vector). However, one significant disadvantage of RF cloning is its low DNA product yield due to linear amplification. In our experience, DNA fragment insertion over 1 kb shows low efficiency and usually calls for the extensive optimization of PCR conditions using RF cloning.

To solve this problem, several methods derived from RF cloning have been established to improve the efficiency of mega primer- and PCR-based cloning. Exponential Megapriming PCR (EMP) cloning [5] and Inverse Fusion PCR cloning (IFPC) [6] obviously improve the size of inserts (up to 2.5 kb) by the introduction of an assistant primer to ensure exponential amplification. However, both methods require phosphorylation and ligation of the final products from the second round of PCR to ensure efficiency, which adds to labour and cost. Another modified version, Circular Polymerase Extension cloning (CPEC) [7], uses two pairs of primers to amplify target DNA and linearize recipient vectors in two separate PCRs. In the second PCR, the insert (single or hybridized) and vector extends using each other as a template until they extend into a circular plasmid with two nicks. These cloning strategies provide alternatives for DNA manipulation and greatly increase the success rate of the construction of recombinant vectors.

Inspired by the cloning methods mentioned above, we present an efficient mega primer-mediated (MP) cloning strategy named as MP cloning, which is amenable to both chimaera construction and long DNA fragment (over 1 kb) insertion. The cloning method requires only two PCR steps: (i) mega primer preparation and (ii) circular amplification. The products of the second PCR are digested by DpnI to eliminate the parent DNA and transformed into competent cells. The entire process can be accomplished within one day. Interestingly, purification of the products of the second PCR can dramatically improve cloning efficiency, although the mechanism is still not clear. As proof-of-concept experiments, using the MP cloning strategy, we successfully constructed pGADT_7_-2000 chimaera (two 1-kb fragments assembly), pcDNA3.1-2000 (four fragments assembly, 0.5 kb each) and pET40-NusA (a 1485-bp fragment insertion). Compared with RF cloning and other derivatives, MP cloning shows a much higher efficiency for longer fragment insertion and fragment assembly.

## Materials and methods

### PCR conditions

All primers were purchased from Sangon (Shanghai, China). The primer sequences are listed in the Supplementary material. High-fidelity DNA polymerase (KOD-Plus-Neo) was purchased from TOYOBO (Osaka, Japan). In the first PCR, DNA fragments were amplified by 20-pmol primers supplemented with 1× reaction buffer, 0.4 mM of dNTPs, 1 mM of MgSO_4_, 1 U of DNA polymerase and 1–50 ng of template DNA in 50 μl of reaction mixtures. Cycling parameters for PCR were 94°C for 2 min, denaturing at 98°C for 10 s, annealing at 60°C for 30 s and elongation at 68°C for 30 cycles (2 kb/min); these steps were followed by a final 10-min extension step. The target fragments were purified by gel purification using a DNA purification kit (TianGen, Beijing, China). In the second PCR, 50–200 ng of purified fragments were used as mega primers and mixed with 1–100 ng of recipient plasmid, which is purified from methylase positive *DH5α* cells, supplemented with 1× PCR Buffer, 0.4 mM of dNTPs, 1 mM of MgSO_4_ and 1 U of DNA polymerase in a 50-μl reaction mixture. Cycling parameters for PCR were 94°C for 2 min, denaturing at 98°C for 10 s, annealing at 60°C for 30 s and elongation at 68°C for 30 cycles (2 kb/min) followed by a final 10-min extension step.

DNA products from the second PCR were purified by a Universal DNA Purification Kit (TianGen, Beijing, China) followed by DpnI digestion. Approximately 8 μl of purified products supplemented with 1 μl of 10× reaction buffer were digested by 1 μl of FastDigest^™^ DpnI (Fermentas, Burlington, Canada) at 37°C for 1 h.

### Transformation, colony PCR and restriction enzyme verificatiion

DNA products were chemically transformed into *DH5α* competent cells (the transformation efficiency is approximately 3 × 10^6^ colonies/μg PUC plasmid), which were produced in our laboratory. All cells were spread on to LB plates containing ampicillin sodium or kanamycin and incubated overnight. Colony PCR was performed using DreamTaq DNA polymerase (Fermentas, Burlington, Canada) according to the manufacturer’s instructions. Further sequence validation by sequencing was performed by Sangon (Shanghai, China). Plasmids were extracted by the TIANprep Mini Plasmid Kit (Tiangen, Beijing, China). Digestion by FastDigest™ NdeI and FastDigest™ XhoI (Fermentas) was performed at 37°C for 1 h.

## Results and discussion

### Chimaeragenesis by MP cloning

Chimaeragenesis is a useful tool for the study of relationships of protein structure and function. For example, by constructing different voltage-gated channel subtypes chimaeras, chimaeragenesis already revealed some important gating properties of the less-characterized sodium channels, such as Na_v_1.9 and Na_v_1.8 [8,9]. Chimaeragenesis, by which two or more DNA modules could be stitched together, is generally achieved by *in vivo* [10] or/and *in vitro* [11] recombination or overlap-extension PCR [12,13]. These methods are greatly useful for chimaeragenesis construction; however, they still have their limitations in barricading HTP-cloning applications. For example, HTP cloning [11] based on the Gateway cloning system utilizes expensive Clonase™ (Invitrogen Corp.) to incorporate fragments carrying *attB1* and *attB2* recombination sites into vectors carrying *attP1* and *attP2* recombination sites. Moreover, the method relies on low efficiency *in vivo* recombination in *E. coli* to form intact recombinant plasmids. Chimaera construction using multiple-template-based sequential PCRs [13] requires extra subcloning steps for assembling fragments into the recipient vector by the restriction enzyme. Chimaeragenesis by MP cloning described here is a more efficient and economical strategy because the target genes can be integrated into the destination vector only by two PCRs rather than by the use of expensive recombination enzymes or multi-cloning steps, and the total task can be accomplished within one day.

**Figure 1:**
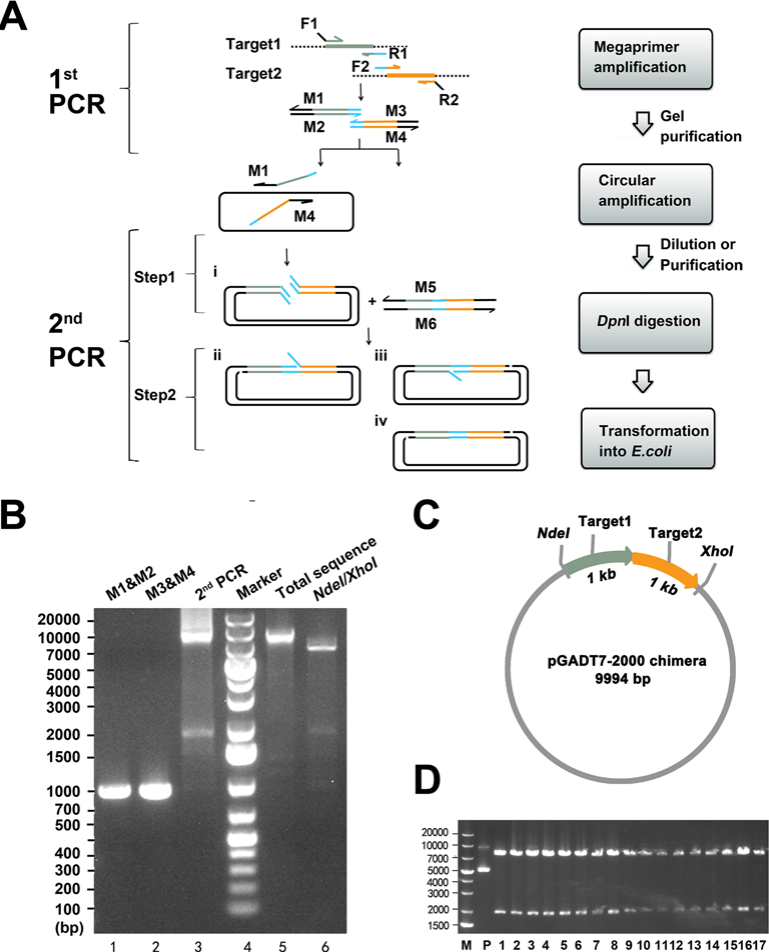
Overview of MP cloning (**A**) Schematic diagram and flowchart of MP cloning. Target gene 1 and target gene 2 were marked grey and orange respectively. The blue part on primer R1 and F2 was homologous with a length of 25 bp derived from target genes (target 1, target 2 or both). M1, M2, M3, M4, M5 and M6 denoted mega primers. (**B**) Construction of pGADT7-2000 chimaera. Lane 1 and lane 2: products (1000 bp) of first PCR, which acted as mega primers (M1 and M2, M3 and M4) in second PCR. Lane 3: products of second PCR. Lane 4: DNA molecular weight marker. Target DNA products were gel purified (lane 5) and identified by NdeI/XhoI (lane 6). (**C**) The map of pGADT7-2000 chimaera. (**D**) Restriction enzyme verification of positive clones by NdeI and XhoI. M: DNA molecular weight marker; P: a pGADT7-2000 chimaera plasmid was used as a control; 1–17: 17 randomly picked clones digested by NdeI and XhoI.

The principle of MP cloning is illustrated in Figure 1A. To construct a chimaera of target gene 1 (target 1) and target gene 2 (target 2) into the destination vector, two pairs of primers were used. The primer pair F1/R1 and the primer pair F2/R2 were used to amplify target 1 and target 2 in the first round of PCR (first PCR) respectively. The 5′-regions of the primers F1 and R2 were homologous to the insertion site in the destination vector with a *T*_m_ of 70°C, and the 3′-regions of the primers F1 and R2 were homologous to the target genes with a *T*_m_ of 70°C. The value of *T*_m_ is calculated according to the formula: *T*_m_ =4(A + T) + 2(G + C). R1 and F2 were internal primers with approximately 25-bp homologous sequences at 5′-end and their 3′-regions were homologous to target genes with a *T*_m_ of 70°C. In the first PCR, the flanked target products were enriched by gel purification individually. The purified products were subsequently utilized as mega primers (M1, M2, M3 and M4) in the second PCR.

Theoretically, two steps were involved in the second PCR. In the first step, M1 and M4 annealed to the destination plasmid and amplified the total sequence exponentially to yield a linear, nicked recombinant plasmid with redundant homologous ends (product i); meanwhile, M2 and M3 annealed to each other and elongated into another pair of mega primers (M5 and M6). In the second step, M5 and M6 annealed to product i and elongated into intact recombinant plasmids with two nicks. In total, four types of productive recombinant vectors (product i, ii, iii and iv) were generated in the second PCR and can be subsequently transformed into *E. coli* competent cells and repaired. However, product i with redundant homologous ends was transformed into *E. coli* less efficiently and circularized by *in vivo* recombination in the RecA-independent pathway with low efficiency [14]. In contrast, the other products (ii, iii and iv) with annealed overhangs are more stable and can be transformed into *E. coli* with much higher efficiency. The template is exponentially amplified in the first step of the second PCR so that the amount of the template plasmid could be very low (1–10 ng), which allows the background to remain at a minimal level.

As a proof-of-concept experiment, two different genes (1000 bp each) were inserted between NdeI and XhoI restriction sites in pGADT_7_ (~7.9 kb, Clontech) by MP cloning to construct a pGADT_7_-2000 chimaera (Figure 1B,C). After the second PCR, the estimated target DNA (~9.9 kb) was gel purified and identified by restriction enzyme digestion (NdeI and XhoI). As expected, corresponding bands were observed at approximately 7.9 kb and 2 kb on the gel.

As shown in Figure 1D, 17 colonies were randomly picked and the recombinant plasmids were purified and identified by restriction enzyme digestion (NdeI/XhoI)*.* All the clones containing the expected 2-kb fragment were identical with the length of the target chimaera. The sequence of the chimaera was further confirmed by DNA sequencing, and the results showed that all the sequenced chimaeras were correct. Because chimaeras consisted of fragments of varied lengths, it is advisable that equal moles of mega primers be used in the second PCR.

**Figure 2:**
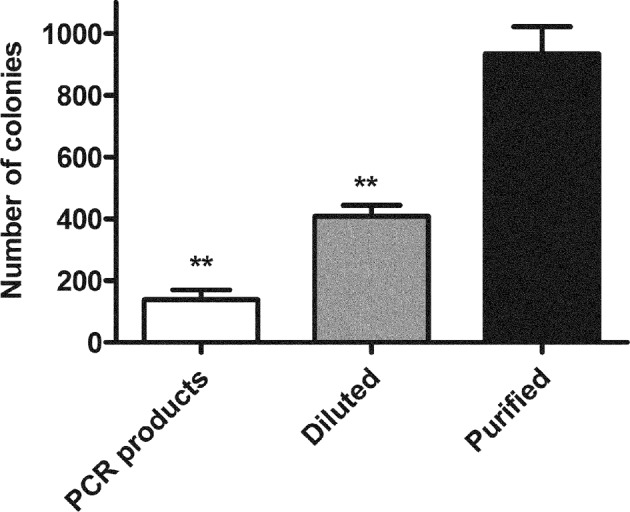
Comparison of cloning efficiency by different modes of processing of DNA products of the second PCR Different treatments of DNA products from the second PCR were compared: directly digested by DpnI (PCR products), diluted followed by DpnI digestion (Diluted) and purified followed by DpnI digestion (Purified). Equal volume (5 μl) of DNA products were transformed into competent cells respectively. The colony number per plate was counted to estimate cloning efficiency. The results are the mean ± S.E.M. of three independent experiments; **P<0.01 compared with Purified.

The products of the second PCR could be directly digested by DpnI to eliminate parental plasmid. Interestingly, the amount of resultant colonies per plate could be significantly enhanced if an additional purification step was performed before DpnI digestion. Dilution of the second PCR products and then direct DpnI digestion could also enhance cloning efficiency (Figure 2). The mechanism is not verified here, but it is speculated because the 3′-exonuclease activity of high-fidelity DNA polymerase during DpnI digestion, which was utilized to develop the FastCloning method [15], can impair the nicked recombinant plasmid and improve cloning efficiency. Although it presents low efficiency, direct digestion may be a preferable choice that can save time and cost and absolutely satisfy routine chimaera construction as well.

Chimaera with a total length of 6000 bp (containing two fragments, 3 kb each) have been successfully constructed by MP cloning, although the cloning efficiency drops with a positive rate of 30% (results not shown). The efficiency might be improved by the application of DNA polymerase with superior processivity and fidelity in the future [16]. The protocol presented here works quite well for chimaeragenesis, although some parameters could be optimized. It was shown that the reaction conditions are quite flexible and compatible, including primer lengths (50–60 bp), PCR (second) cycles (25–30), annealing temperature (55–70°C), the amount of mega primers (50–200 ng) applied to the second PCR and the template plasmid in the second PCR (1–100 ng). It should be noted that an inherent limitation of the PCR-based cloning is that a mutation may be introduced into the constructs during elongation even if high-fidelity DNA polymerase was used; thus, sequencing for confirmation of the correct target genes is still required.

**Figure 3:**
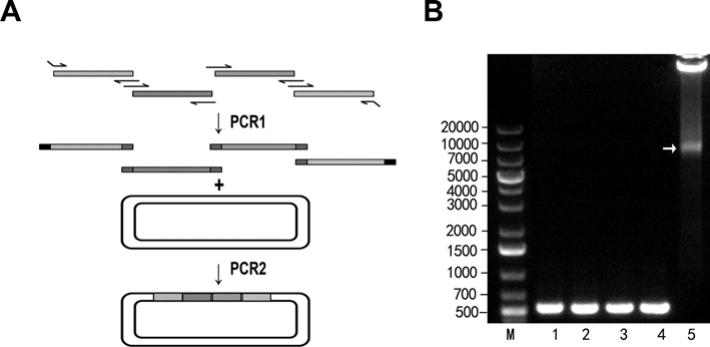
MP cloning for multi-fragment assembly (**A**) Schematic diagram for multi-fragment assembly. (**B**) Four 500-bp fragments were assembled in the second round of PCR. The target band is denoted by an arrow.

In addition to two-fragment chimaeragenesis, we asked whether more DNA fragments could be assembled by MP cloning. Four 500-bp fragments derived from rNa_v_1.4 (GenBank: Y17153.1) were amplified and purified as mega primers (Figure 3). In the second PCR, the mega primers took the vector as a template and amplified, and the four fragments were assembled into the plasmid vector pCDNA3.1 (see ‘Materials and Methods’ section for details). Until now, the MP cloning strategy worked well for an assembly of four short fragments (<500 bp). The number and sizes of the fragments assembled by MP cloning are still to be validated.

### Long DNA fragment insertion/replacement by MP cloning

**Figure 4:**
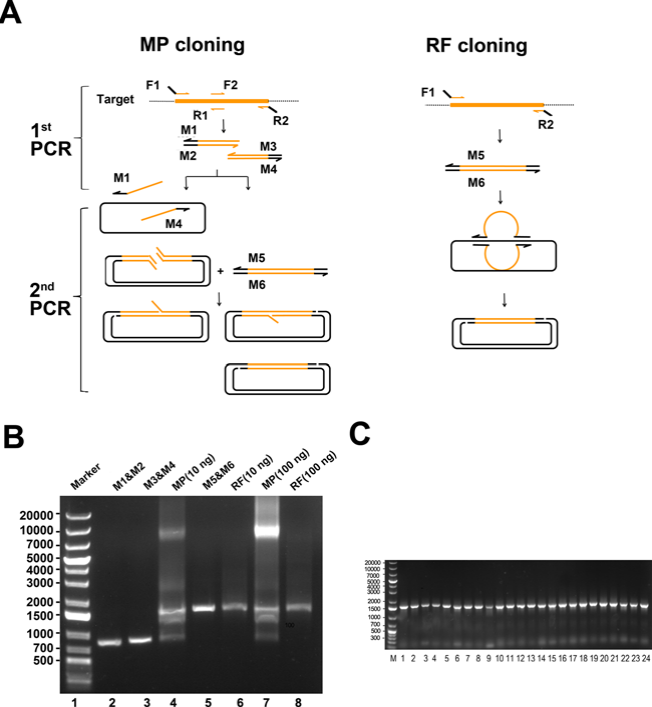
DNA fragments insertion by MP cloning (**A**) Schematic comparison of MP cloning and RF cloning. (**B**) Target DNA yields by MP and RF. Lane 1: DNA molecular weight marker. By MP cloning, the target gene was dissociated into two parts (lane 2 and lane 3) and amplified. The two parts acted as mega primers in the second PCR (lane 4, in which the 10-ng template plasmid was used and lane 7, in which the 100-ng template DNA was used). For RF cloning, the target was amplified by F1 and R2 and used as a mega primer in the second PCR (lane 6, in which the 10-ng template was used, and lane 6 in which the 100-ng template was used). (**C**) Screening by colony PCR. M: DNA molecular weight marker; 1–24: 24 randomly picked colonies identified by PCR. The length of inserts was 1500 bp.

Using the same principle as chimaeragenesis, MP cloning can also be applied to seamlessly clone a single DNA fragment (especially longer than 1 kb) into the vector. Comparison with another widely used seamless cloning method (RF cloning), it was proven that the MP cloning is more effective for longer fragment insertion. Compared with RF cloning (Figure 4A), which only needs F1 and R2 primers, MP cloning needs two extra middle primers (R1 and F2). In the first PCR, using MP cloning, the target DNA fragment is divided into two parts by two pairs of primers, which are used as mega primers in the following PCR. The two pairs of mega primers can exponentially amplify destination plasmids in the second PCR rather than linearly amplify them when using RF cloning, which greatly improves the success rate of long-fragment insertion. To the best of our knowledge, RF cloning can work well for fragments shorter than 1000 bp, whereas the efficiency of another OEC is also reduced as the length of inserts increases [17], and the target band cannot always be detected on the gel electrophoresis assay due to the linear amplification of the plasmid [5]. Moreover, for RF cloning, larger fragments might not anneal to the destination vector efficiently because of the very long unmatched portion (the loop portion, Figure 4A) flanked by F1 and R2 constitutes a hindrance.

To insert a 1485-bp gene into the pET-40b vector (6190 bp, Novagen), RF cloning and MP cloning were used and compared (Figure 4B). Interestingly, the target band could still be detected by MP cloning using the template plasmid if the amount was 10 ng in the second PCR; in contrast, using RF cloning, the band was hardly detected by gel electrophoresis even when a template plasmid up to 100 ng was used. Still, purification of the products of the second PCR ahead of DpnI digestion could dramatically increase the resultant colonies compared with the unpurified group. Thousands of colonies per plate could be harvested by MP cloning, whereas only 15 colonies appeared on the plate by RF cloning; this finding is even the case when a template plasmid up to 100 ng was used in the second PCR. Twenty-four colonies were randomly selected by MP cloning and identified by bacterial colony PCR and all the clones were positive (Figure 4C). The MP cloning we developed showed higher efficiency and is more suitable for longer fragment insertion than RF cloning.

## Conclusion

One drawback of MP cloning is that mutation(s) may be introduced during elongation. However, in our case, mutations introduced by DNA polymerases are rarely observed. Moreover, with the improvement of high-fidelity DNA polymerases and less PCR cycles, the problem can be well handled. It is expected that the MP cloning might be developed to a HTP cloning tool to satisfy the needs of the rapid increase in functional genomic studies and protein engineering.

## Abbreviations

HTP: high-throughput
MP: mega primer-mediated
OEC: overlap-extension cloning
RF: restriction-free

## References

[1] Lu Q. (2005) Seamless cloning and gene fusion. Trends Biotechnol. 23, 199–207 15780712 10.1016/j.tibtech.2005.02.008 PMC7119129

[2] Tillett D. and Neilan B.A. (1999) Enzyme-free cloning: a rapid method to clone PCR products independent of vector restriction enzyme sites. Nucleic Acids Res. 27, e26 10481038 10.1093/nar/27.19.e26 PMC148636

[3] van den Ent F. and Löwe J. (2006) RF cloning: a restriction-free method for inserting target genes into plasmids. J. Biochem. Bioph. Methods 67, 67–74 10.1016/j.jbbm.2005.12.008 16480772

[4] Bryksin A.V. and Matsumura I. (2010) Overlap extension PCR cloning: a simple and reliable way to create recombinant plasmids. Biotechniques 48, 463–465 20569222 10.2144/000113418 PMC3121328

[5] Ulrich A., Andersen K.R. and Schwartz T.U. (2012) Exponential Megapriming PCR (EMP) cloning—seamless DNA insertion into any target plasmid without sequence constraints. PLoS ONE 7, e53360 23300917 10.1371/journal.pone.0053360 PMC3534072

[6] Spiliotis M. (2012) Inverse fusion PCR cloning. PLoS ONE 7, e35407 22530019 10.1371/journal.pone.0035407 PMC3328455

[7] Quan J. and Tian J. (2011) Circular polymerase extension cloning for high-throughput cloning of complex and combinatorial DNA libraries. Nat. Protoc. 6, 242–251 21293463 10.1038/nprot.2010.181

[8] Marques M.R., Mendes M.A., Tormena C.F., Souza B.M., Ribeiro S.P., Rittner R. (2004) Structure determination of an organometallic 1-(diazenylaryl)ethanol: a novel toxin subclass from the web of the spider *Nephila clavipes*. Chem. Biodivers. 1, 830–838 17191883 10.1002/cbdv.200490065

[9] Meng E., Cai T.F., Zhang H., Tang S., Li M.J., Li W.Y. (2014) Screening for voltage-gated sodium channel interacting peptides. Sci. Rep. 4, 4569 24691553 10.1038/srep04569 PMC3972499

[10] Escoubas P., Diochot S. and Corzo G. (2000) Structure and pharmacology of spider venom neurotoxins. Biochimie 82, 893–907 11086219 10.1016/s0300-9084(00)01166-4

[11] Sogin M.L. and Silberman J.D. (1998) Evolution of the protists and protistan parasites from the perspective of molecular systematics. Int. J. Parasitol. 28, 11–20 9504331 10.1016/s0020-7519(97)00181-1

[12] Corzo G., Sabo J.K., Bosmans F., Billen B., Villegas E., Tytgat J. (2007) Solution structure and alanine scan of a spider toxin that affects the activation of mammalian voltage-gated sodium channels. J. Biol. Chem. 282, 4643–4652 17148449 10.1074/jbc.M605403200

[13] Boyd W.C. (1949) Systematics, evolution, and anthropology in the light of immunology. Q. Rev. Biol. 24, 102–108 18153122 10.1086/396941

[14] Zhang Y., Werling U. and Edelmann W. (2012) SLiCE: a novel bacterial cell extract-based DNA cloning method. Nucleic Acids Res. 40, e55 22241772 10.1093/nar/gkr1288 PMC3333860

[15] Li C., Wen A., Shen B., Lu J., Huang Y. and Chang Y. (2011) FastCloning: a highly simplified, purification-free, sequence- and ligation-independent PCR cloning method. BMC Biotechnol. 11, 92 21992524 10.1186/1472-6750-11-92 PMC3207894

[16] You C., Zhang X.Z. and Zhang Y.H.P. (2011) Simple cloning via direct transformation of PCR product (DNA M\multimer) to *Escherichia coli* and *Bacillus subtilis*. Appl. Environ. Microbiol. 78, 1593–1595 22194286 10.1128/AEM.07105-11 PMC3294473

[17] Stevenson J., Krycer J.R., Phan L. and Brown A.J. (2013) A practical comparison of ligation-independent cloning techniques. PLoS ONE 8, e83888 24376768 10.1371/journal.pone.0083888 PMC3871625

